# Synovial fluid and plasma concentrations of tedizolid in patients with osteoarthritis infected with *Staphylococcus aureus* effectively determined with fluorescence detection

**DOI:** 10.1186/s40780-023-00303-z

**Published:** 2023-10-10

**Authors:** Daisuke Negishi, Okimichi Mitsumatsu, Hiromi Mitsumatsu, Miaki Makiguchi, Makiko Shimizu, Hiroshi Yamazaki

**Affiliations:** 1 Laboratory of Drug Metabolism and Pharmacokinetics, Showa Pharmaceutical University, 3-3165 Higashi-tamagawa Gakuen, Machida, Tokyo, 194-8543 Japan; 2 Kamakura Hospital, 1-8 Hase 3-chome, Kamakura, 248-0016 Kanagawa Japan

**Keywords:** Drug monitoring data, Osteoarthritis, Synovial fluid penetration

## Abstract

**Background:**

Tedizolid is a new oxazolidinone antibiotic with high potency for the treatment of infections caused by methicillin-resistant *Staphylococcus aureus* and other species.

**Case presentation:**

Two patients with osteoarthritis (women aged 79 and 73 years, cases 1 and 2, respectively) infected with *S. aureus* were successfully treated with tedizolid after administration of 200 mg once daily via intravenous infusion. The synovial fluid and plasma concentrations of tedizolid during surgery in case 1 at day 7 were 2.1 and 1.6 µg/mL, respectively, yielding a ratio of synovial fluid/plasma of 130%. Those in case 2 at day 2 were 2.9 and 3.3 µg/mL, respectively, corresponding to a ratio of synovial fluid/plasma of 88%.

**Conclusions:**

These results imply very similar concentrations of tedizolid in the synovial fluid and plasma of osteoarthritis patients with acute *S. aureus* infection.

## Background

Tedizolid is a novel oxazolidinone antibiotic prodrug used to treat infections caused by gram-positive organisms such as methicillin-resistant *Staphylococcus aureus* [1–3]. Tedizolid phosphate is a promising antibiotic; however, its use is restricted by the adverse effects of long-term administration of 200 mg once daily [4–6]. Bronchoalveolar lavage, lung epithelial lining fluid, cerebrospinal fluid, skin, and soft tissue concentrations of tedizolid following a single dose in healthy volunteers [5–9] and rats [10], and the population pharmacokinetics of tedizolid in healthy subjects [11], have been reported.

Pharmacokinetic and tissue penetration data for tedizolid in hospitalized patients with foot infections have been reported [8]. However, it is unknown whether synovial fluids contain sufficient concentrations of tedizolid delivered from the plasma of patients with osteoarthritis. From a clinical perspective, the monitoring of synovial fluid concentrations of tedizolid is of interest in comparison with plasma pharmacokinetics. Against this background, in the present study, two patients with acute osteoarthritis infection were successfully treated with tedizolid. Synovial fluid was sampled during surgery in these two patients; subsequently, the concentrations of tedizolid were determined and the synovial fluid/plasma ratios were calculated and evaluated.

## Case presentation

**Fig. 1:**
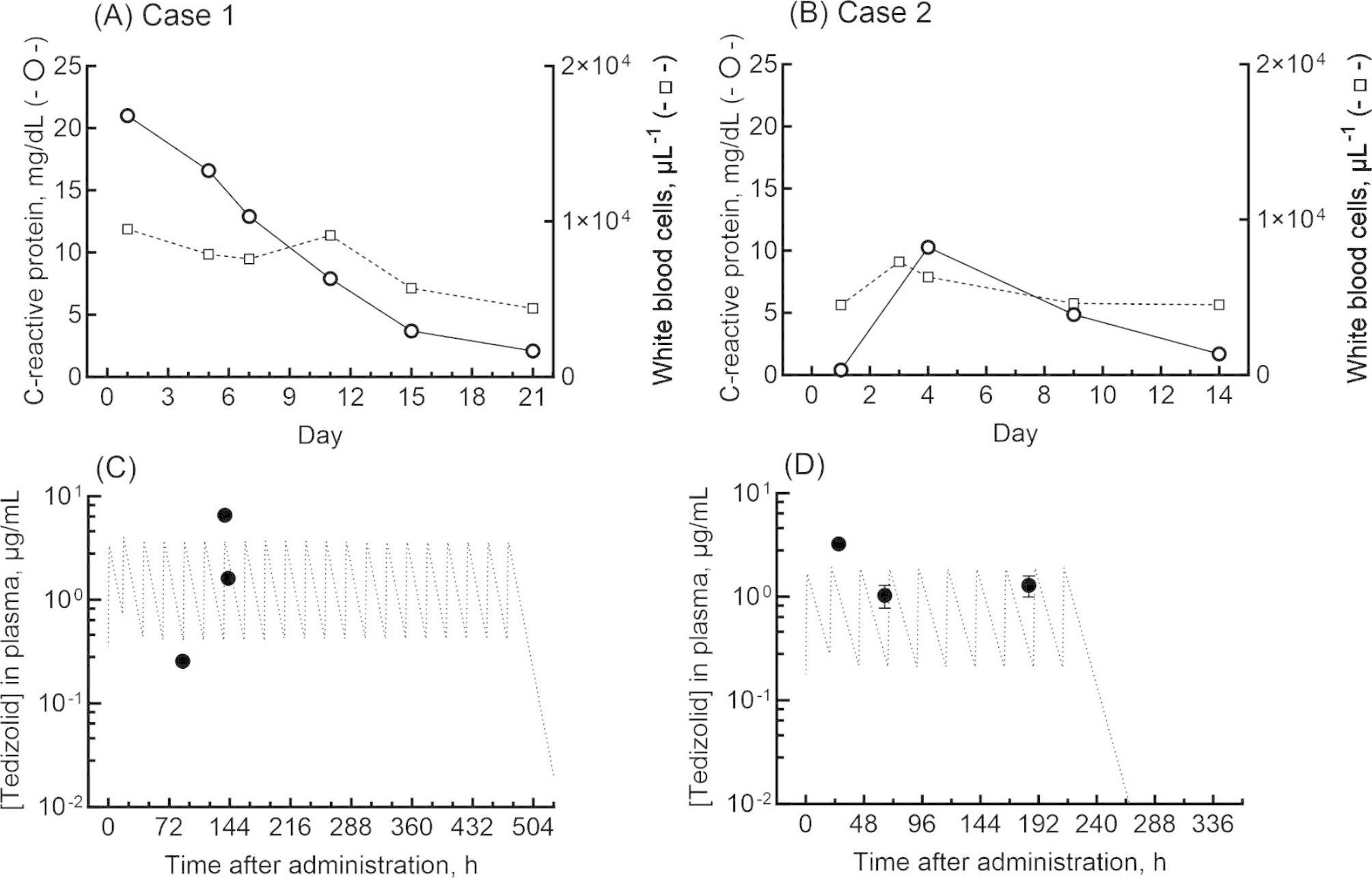
Clinical laboratory results and measured plasma concentrations of tedizolid (solid circles) in two patients administered 200 mg once daily via intravenous infusion. Levels are shown of C-reactive protein (circles, mg/dL) and white blood cell counts (squares, µL^− 1^) in the two patients: (**A**) a 79-year-old woman (48 kg), case 1, and (**B**) a 73-year-old woman (80 kg), case 2. The measured plasma concentrations of tedizolid (solid circles) in cases 1 (**C**) and 2 (**D**) are shown after once-daily administrations. Plasma concentrations of tedizolid (dashed lines) after virtual daily doses were generated using a previously reported one-compartment model [11], using Microsoft Excel

Approval for the current study was given by the Ethics Committee of Kamakura Hospital. Two patients with acute osteoarthritis were successfully treated with tedizolid between April 2023 and June 2023. The plasma and synovial fluid concentrations of tedizolid in these two patients (women aged 79 and 73 years, Table 1), who were treated at Kamakura Hospital for osteoarthritis infected with *S. aureus*, were evaluated after administration of 200 mg once daily by intravenous infusion (at 10 am) for 7 days in case 1 and for 2 days in case 2. The patients provided written informed consent to participate in this study and for its publication. The clinical laboratory results of these two patients over a 2- or 3-week period are shown in Fig. 1A and B. Clinical laboratory data before tedizolid treatment for case 1 were plasma creatinine (0.91 mg/dL), plasma bilirubin (1.6 mg/dL), and platelets (17.1 × 10^4^/µL), whereas those for case 2 were plasma creatinine (0.80 mg/dL), plasma bilirubin (0.6 mg/dL), and platelets (26.6 × 10^4^/µL). Drugs co-administered (per day) during tedizolid treatment in case 1 were candesartan (4 mg), dapagliflozin (5 mg), vildagliptin (100 mg), metformin (1000 mg), and pitavastatin (2 mg), whereas those in case 2 were telmisartan (40 mg), amlodipine (5 mg), loxoprofen (180 mg), lansoprazole (15 mg), and pitavastatin (1 mg). The patients were found to have methicillin-sensitive *S. aureus* in synovial fluids by outsourced clinical laboratory services.

**Table 1:** Plasma and synovial fluid concentrations of tedizolid determined in two patients during surgery

Case (body weight)		Sampling	Tedizolid concentration (µg/mL)		Ratio of synovial fluid/plasma
			Plasma	Synovial fluid	
1	79-year-old woman (48 kg)	Day 7	1.6 ± 0.1	2.1 ± 0.1	130%
2	73-year-old woman (80 kg )	Day 2	3.3 ± 0.2	2.9 ± 0.4	88%

Data are presented as the mean ± standard deviation from triplicate determinations

**Fig. 2:**
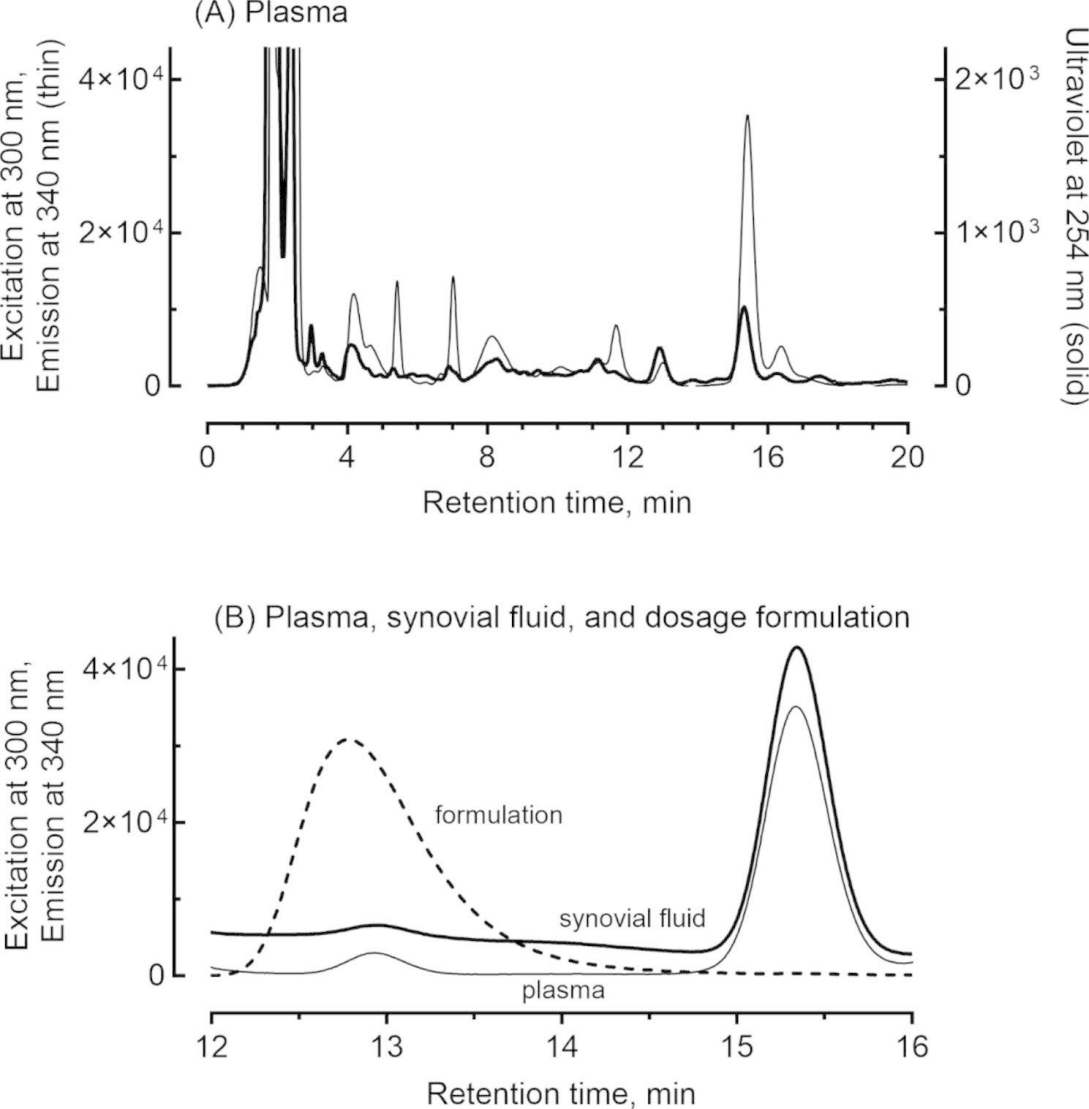
Representative chromatograms for tedizolid in plasma and synovial fluid. (**A**) The patient in case 1 was treated with tedizolid once a day; her plasma samples were analyzed using fluorescence (thin line) and ultraviolet (solid line) detectors. (**B**) Tedizolid levels in samples of plasma (thin line) and synovial fluid (solid line) were determined using a fluorescence detector. The retention times of tedizolid and tedizolid phosphate derived from a dosage formulation were 15.3 and 12.9 min, respectively

The tedizolid levels in the plasma and synovial fluid samples from the two patients at 5 and 7 h after the last administration on Days 7 and 2, respectively (Fig. 1), were quantified using liquid chromatography (Fig. 2)

Samples of synovial fluid were obtained from both patients during surgery or knee aspiration, and these, in addition to plasma samples, were analyzed pharmacokinetically. In the preliminary study, plasma samples from two additional patients (an 84-year-old woman, 49 kg, and an 80-year-old woman, 54 kg) were analyzed. Samples (100 µL) of plasma and synovial fluid obtained from the four patients were mixed with an equivalent volume of acetonitrile, and the aqueous supernatant was centrifuged at 15,000 × *g* for 10 min at 4 °C. Synovial fluid and plasma levels of tedizolid were measured using a reversed-phase, high-performance, liquid chromatography system (Shimadzu, Kyoto, Japan) with a Mightysil RP-18GP Aqua column (5 μm, 150 × 4.6 mm, Kanto Chemical, Tokyo, Japan) equilibrated in a mobile phase comprising 35% CH_3_CN in 0.1% aqueous phosphoric acid at a flow rate of 1.0 mL/min and a column temperature of 45 °C. Fluorescence monitoring was carried out at excitation and emission wavelengths of 300 and 340 nm [12], respectively, and ultraviolet monitoring was done at 254 nm. The samples (10 µL) were infused using an autosampler. Figure 2 demonstrates typical chromograms of tedizolid in plasma and synovial fluid. The retention time of tedizolid was 15.3 min. For cases 1 and 2, the determined plasma concentrations of tedizolid and the concentration profiles generated using a pharmacokinetic model [set up from a previously described one-compartment model that used the following parameters: a common half-life of 7.1 h (calculated from 6.69 L/h of clearance and median volume of distribution of 69 L), and individual volumes of distributions of 46 and 91 L, respectively, for the body weights of cases 1 and 2 (calculated from 52 L for 5th percentile of 52 kg, 69 L for median of 65 kg, and 88 L for 95th percentile of 78 kg)] are shown in Fig. 1C and D, respectively [11]. The concentrations of tedizolid in plasma samples obtained during surgery for case 1 on day 7 and for case 2 on day 2 were 1.6 and 3.3 µg/mL, respectively. Synovial fluid levels of tedizolid obtained during surgery in case 1 and case 2 were 2.1 and 2.9 µg/mL, respectively; these concentrations represented synovial fluid/plasma ratios of 130% and 88% (Table 1). Data are presented as the mean from triplicate determinations. The biological matrix did not affect the measured concentrations.

## Discussion and conclusion

Validated, simple, or enantio-separation of tedizolid using high-performance liquid chromatography has been proposed with tandem mass spectrometry, ultraviolet, or fluorescence detection [12, 13]. Relatively good penetration of tedizolid into the interstitial fluid of adipose and muscle tissues has been demonstrated in healthy volunteers [9], whereas inadequate plasma-to-cerebrospinal fluid ratios of 54% have been reported for tedizolid [7]. In the current study, plasma concentrations of tedizolid in the two patients were similar to those in synovial fluid, yielding synovial fluid/plasma ratios of 88–130%. Successful treatment of osteoarthritis infected with *S. aureus* was confirmed after the patients were administered multiple 200-mg doses (as once-daily intravenous infusions) of tedizolid phosphate; the dose was set using a dosage formulation. A population-based pharmacokinetic model of tedizolid in hospitalized patients with acute arthritis has been proposed [11]. Reportedly, only body weight and total bilirubin levels were covariates of the pharmacokinetic model parameters for tedizolid, and therefore were likely to have clinically relevant effects on tedizolid exposure; moreover, the platelet count was found not to be a covariate [11]. It should be noted that the plasma concentrations of tedizolid determined in this study and the values estimated using the one-compartment model with reported population pharmacokinetics [11] were mostly consistent (Fig. 1C and D). The only drug co-administrated in common during the treatment in both cases was pitavastatin, which is a well-known hepatic organic anion transporting polypeptide substrate (OATPs) [14]. Possible drug interactions with tedizolid and pitavastatin may be involved in the relatively high plasma concentrations of tedizolid in the two cases analyzed here; however, the roles of OATPs in the apparently high exposure of tedizolid could be ruled out based on the negative findings of the interview form (package insert) of this medicine. In case 1, the apparently high plasma concentration of tedizolid might have resulted from the high plasma bilirubin concentration (1.6 mg/dL), because bilirubin is one of the clinically relevant covariates [11]. In our preliminary determinations, plasma concentrations of tedizolid in the two additional cases 20 h after the last administration were 0.6 and 0.9 µg/mL, a similar finding to those of cases 1 and 2, as described above. Although tedizolid is a novel oxazolidinone antibiotic prodrug used to treat infections caused by gram-positive organisms, such as methicillin-resistant *S. aureus* [1–3], the hospital director and attending physicians of patients with osteoarthritis in the cases decided to use this medicine for medical reasons (pretreatments, surgeries during hospitalization, etc.) before the outsourced test results in terms of methicillin resistance.

In conclusion, simple systematic therapeutic drug monitoring of tedizolid, which may indicate equivalent concentrations of tedizolid in the synovial fluid after multiple administrations, could have a positive clinical impact on the treatment of infections of the synovial fluid in osteoarthritis patients.

## Data Availability

All data generated or analyzed during this study are included in this published article and are also available from the corresponding author upon reasonable request.
